# Comparative Humoral Immune Responses Induced by Live-Attenuated and Inactivated Porcine Epidemic Diarrhea Vaccines in Replacement Gilts

**DOI:** 10.3390/vaccines14030231

**Published:** 2026-02-28

**Authors:** Prapassorn Boonsoongnern, Orawan Boodde, Wilairat Chumsing, Pichai Jirawattanapong, Manakorn Sukmak, Yonlayong Woonwong, Narut Thanantong, Worawidh Wajjwalku, Alongkot Boonsoongnern

**Affiliations:** 1 Department of Anatomy, Faculty of Veterinary Medicine, Kasetsart University, Bangkok 10900, Thailand; prapassorn.j@ku.th; 2 Department of Farm Resources and Production Medicine, Faculty of Veterinary Medicine, Kasetsart University, Nakhon Pathom 73140, Thailand; fvetorb@ku.ac.th (O.B.); fvetwich@ku.ac.th (W.C.); pichai.j@ku.th (P.J.); manakorn.s@ku.th (M.S.); yonlayong.w@ku.th (Y.W.); narut.t@ku.th (N.T.); 3 Akkharatchakumari Veterinary College, Walailak University, Nakhon Si Thammarat 80160, Thailand; worawidh.wa@wu.ac.th

**Keywords:** PEDV, live-attenuated PEDV vaccine, inactivated PEDV vaccine, heterologous prime-boost, replacement gilts, humoral immunity, neutralizing antibody

## Abstract

**Background/Objectives**: Porcine epidemic diarrhea (PED) is a highly contagious enteric disease caused by porcine epidemic diarrhea virus (PEDV) and is associated with severe clinical signs and high mortality in neonatal piglets. Vaccination is an important strategy for PED control through the induction of humoral immunity. This study aimed to compare immune responses induced by inactivated and live-attenuated PEDV vaccines and to evaluate a heterologous prime-boost vaccination strategy in PEDV-naïve replacement gilts. **Methods**: Twenty-four PEDV-naïve replacement gilts were randomly assigned to four groups: unvaccinated control, inactivated vaccine administered twice (K/K), live-attenuated vaccine administered twice (L/L), and live-attenuated priming followed by an inactivated booster (L/K). Pigs received two intramuscular vaccinations at 16 weeks of age and two weeks later. Serum samples collected up to 42 days post-vaccination were analyzed for PEDV-specific IgG and IgA antibodies by ELISA, and serum-neutralizing antibody titers were determined using a serum neutralization test. **Results**: The L/K regimen induced the highest PEDV-specific IgG responses, with peak levels at day 28 post-vaccination that were significantly higher than those in the K/K and control groups. Serum-neutralizing antibody titers were significantly higher in the L/K and L/L groups than in the K/K and control groups. Serum IgA responses were low and transient across all vaccination groups. **Conclusions**: A heterologous prime-boost vaccination strategy using a live-attenuated PEDV vaccine followed by an inactivated booster induces strong systemic humoral immune responses in replacement gilts and represents a promising approach for PEDV vaccination programs.

## 1. Introduction

Porcine epidemic diarrhea (PED) is a highly contagious enteric disease of swine caused by porcine epidemic diarrhea virus (PEDV), an enveloped, positive-sense, single-stranded RNA virus belonging to the genus *Alphacoronavirus* [1,2,3]. PEDV continues to circulate globally and remains a major economic threat to the swine industry, primarily due to severe watery diarrhea, dehydration, and high mortality in neonatal piglets [4,5,6]. Antigenic drift within the spike (S) glycoprotein has contributed to recurrent outbreaks and the emergence of variant strains with altered virulence and immune recognition [7,8,9,10]. Consequently, vaccination strategies capable of inducing robust and durable immune responses against PEDV remain a critical component of swine health management.

Despite the availability of commercial PEDV vaccines, their protective efficacy under field conditions has been reported to vary across regions and production systems [11]. One important factor contributing to this variability is antigenic divergence between classical vaccine strains and currently circulating epidemic field isolates, particularly within the spike (S) glycoprotein, the principal target of virus-neutralizing antibodies. Experimental challenge studies have demonstrated reduced cross-protective efficacy of classical G1a strains against heterologous G2a epidemic strains, indicating that genotype differences within the spike gene can compromise vaccine-induced immunity [12]. Furthermore, molecular epidemiological analyses based on spike gene sequences have revealed substantial genetic diversity and ongoing viral evolution among PEDV strains circulating in Thailand [13,14], emphasizing the importance of regionally relevant vaccine evaluation. Together, these findings highlight the need to systematically assess vaccine regimens that are antigenically matched to locally circulating strains and optimized for specific production contexts.

Because neonatal piglets rely predominantly on passive lactogenic immunity, high levels of neutralizing antibodies, particularly IgA in the colostrum and milk of pregnant sows, are recognized as the primary correlates of protection against PEDV under field conditions [15,16,17]. Although feedback exposure has been applied in some regions, including Thailand, to stimulate herd immunity, this practice carries substantial biosafety risks, such as co-transmission of other pathogens and the potential selection of antigenic variants [18,19,20]. Therefore, the development and optimization of safe and effective PEDV vaccination strategies remain essential for disease prevention and control.

Protection against enteric coronaviruses such as PEDV is largely mediated by lactogenic immunity, whereby antibodies produced in the dam are transferred to suckling piglets through colostrum and milk and provide protection during early life [21,22]. This mechanism is particularly important for PEDV, as neonatal piglets possess an immature immune system and depend almost entirely on maternally derived antibodies to mitigate severe enteric disease [22,23]. Although lactogenic immunity represents the ultimate correlate of protection in the field, systemic humoral immune responses—including serum IgG and functional neutralizing antibodies—are widely used as surrogate indicators of vaccine-induced immune priming prior to evaluation in pregnant sows [21,24]. While serum IgA may provide supportive information on humoral activation, evaluation of systemic antibodies in replacement gilts serves as an indicator of immune priming, whereas definitive assessment of protective lactogenic immunity requires analysis of mucosal antibody responses in pregnant sows.

Vaccine-induced immunity is influenced by the nature of the antigen, the adjuvant, and the immunization schedule. Live-attenuated vaccines are known to elicit strong innate and adaptive immune activation, whereas inactivated vaccines generally provide a safer but sometimes less potent immunogenic stimulus [25,26,27]. Serial passaging of PEDV in cell culture has been employed to attenuate viral virulence and generate injectable live-attenuated vaccine candidates [28]. In this context, heterologous prime-boost vaccination strategies combining live-attenuated and inactivated PEDV vaccines have been shown to enhance the magnitude, breadth, and durability of humoral immune responses compared with homologous regimens [29].

Although pregnant sows are the primary target population for PEDV vaccination, replacement gilts represent a practical and ethically feasible pre-breeding model for evaluating systemic vaccine-induced immune priming, including serum IgG and neutralizing antibody responses, before advancing to studies in pregnant animals [30,31]. Parenteral vaccination in pigs predominantly induces systemic humoral immunity, whereas mucosal IgA responses are limited due to compartmentalization of the mucosal immune system [22], highlighting the relevance of assessing both systemic antibody responses and functional neutralizing activity in this model.

This study aimed to compare humoral immune responses, including PEDV-specific IgG, IgA, and serum-neutralizing antibody titers, induced by inactivated and live-attenuated PEDV vaccines administered as homologous or heterologous prime-boost regimens in PEDV-naïve replacement gilts. The findings are intended to support rational vaccine program selection and optimization of PEDV control strategies in sow herds.

## 2. Materials and Methods

### 2.1. Viruses and Cells

PEDV strain TRANG/37, Thailand (GenBank accession no. MN379926) was propagated in Vero cells (ATCC^®^ CCL-81™). Vero cells were maintained in Dulbecco’s Modified Eagle Medium (DMEM; Gibco, Grand Island, NY, USA) supplemented with 5% heat-inactivated fetal bovine serum (FBS), 100 U/mL penicillin, and 100 µg/mL streptomycin at 37 °C in a humidified 5% CO_2_ incubator. For virus propagation, confluent monolayers were washed with PBS and inoculated with PEDV at a multiplicity of infection (MOI) of 0.1 in serum-free DMEM containing 10 µg/mL trypsin. After adsorption for 1 h, fresh infection medium was added. Cultures were incubated at 37 °C and monitored daily for cytopathic effects (CPE). Under our experimental conditions, approximately 80% CPE was consistently observed at 4 days post-inoculation (dpi), at which point the cultures were harvested. The incubation period was strictly limited to 4 days to minimize potential age-related degeneration of the cell monolayer. Mock-infected control cultures were maintained in parallel to distinguish virus-induced CPE from non-specific cellular changes. Virus-containing supernatants were then harvested, clarified by centrifugation, aliquoted, and stored at −80 °C until use.

### 2.2. Vaccine Preparation

Two types of PEDV vaccines, live-attenuated and inactivated (killed), were prepared for use in this study. For the live-attenuated vaccine, PEDV TRANG/37 was serially passaged 150 times in Vero cells to achieve attenuation. Serial passage in Vero cells is a classical approach for viral attenuation. The attenuated phenotype of the passage 150 virus was verified based on stable replication in Vero cells and the absence of clinical signs (fever, diarrhea, or reduced appetite) in vaccinated pigs following immunization. Viral supernatant from passage 150, containing approximately 1 × 10^5^ TCID_50_/mL, was mixed with 10% (*v*/*v*) Montanide™ Gel 01 adjuvant (Seppic, Paris, France) to formulate the final vaccine. Each pig received 2 mL per dose, corresponding to approximately 2 × 10^5^ TCID_50_ of live PEDV per immunization.

For the inactivated vaccine, PEDV was propagated to passage 20 and harvested at approximately 1 × 10^5^ TCID_50_/mL. Viral suspensions were chemically inactivated using 0.1 M binary ethylenimine (BEI) at 37 °C for 48 h, followed by neutralization with 10% sodium thiosulfate. Complete inactivation was confirmed by three blind passages in Vero cells with no detectable cytopathic effect. The inactivated antigen was then formulated with 10% Montanide™ Gel 01 to produce the final vaccine, and each pig received 2 mL per dose, corresponding to approximately 2 × 10^5^ TCID_50_ equivalents per immunization.

### 2.3. Animals, Experimental Design, and Sample Collection

Twenty-four clinically healthy two-way crossbred commercial replacement gilts (Large White × Landrace), 16 weeks of age, were obtained from a commercial PEDV-free farm in Thailand. The herd was routinely monitored and confirmed to be free from porcine epidemic diarrhea virus (PEDV), porcine reproductive and respiratory syndrome virus (PRRSV), porcine circovirus type 2 (PCV2), classical swine fever virus (CSFV), and African swine fever virus (ASFV). None of the gilts had a history of PEDV vaccination. Prior to the experiment, serum samples were tested by enzyme-linked immunosorbent assay (ELISA) and real-time reverse transcription PCR (RT-PCR) to confirm their PEDV-negative status.

Pigs were randomly allocated to four groups (*n* = 6 per group):

1. Control: PBS (unvaccinated).
2. K/K: inactivated vaccine prime + inactivated vaccine boost.
3. L/L: live-attenuated vaccine prime + live-attenuated vaccine boost.
4. L/K: live-attenuated vaccine prime + inactivated vaccine boost.

All pigs were vaccinated intramuscularly at 16 weeks of age (day 0) and boosted 2 weeks later (day 14). Blood samples were collected on days 0, 7, 14, 21, 28, 35, and 42 post-primary vaccination. All pigs were observed daily for general health status following vaccination, including changes in appetite, behavior, fecal consistency, and local injection site reactions.

All animal procedures were approved by the Institutional Animal Care and Use Committee, Faculty of Veterinary Medicine, Kasetsart University, Thailand (ACKU63-VET-003), and followed ARRIVE guidelines.

### 2.4. Enzyme-Linked Immunosorbent Assay (ELISA)

Anti-PEDV IgG and IgA antibodies in serum were measured using an in-house indirect ELISA previously developed and validated in our laboratory. PEDV was propagated in Vero cells, and virus-containing supernatant was clarified by centrifugation at 4000× *g* for 15 min at 4 °C to remove cell debris. The virus was then concentrated by high-speed centrifugation at 13,000× *g* for 3 h at 4 °C, and the resulting pellet was washed twice with sterile phosphate-buffered saline (PBS, pH 7.4), resuspended in PBS, and stored at −80 °C until use. The viral antigen was chemically inactivated prior to plate coating.

Polystyrene 96-well plates (Nalge Nunc Corp., Rochester, NY, USA) were coated with 100 µL per well of viral antigen diluted in 0.5 M carbonate–bicarbonate buffer (pH 9.6) at the previously optimized coating dilution, corresponding to a final coating amount of 312 ng per well, as determined by checkerboard titration during assay validation [32], and incubated overnight at 4 °C. Plates were washed three times with 0.01% Tween-20 in PBS (PBST) and blocked with 150 µL of 5% skim milk in PBS at 37 °C for 1 h. After washing, serum samples diluted 1:40 (based on prior optimization by checkerboard titration) were added in duplicate (100 µL per well) and incubated at 37 °C for 1 h. Plates were washed and incubated with HRP-conjugated anti-swine IgG (KPL, Gaithersburg, MD, USA; 1:10,000) or HRP-conjugated anti-swine IgA (Thermo Fisher Scientific, Waltham, MA, USA; 1:4000) diluted in 1% skim milk/PBS at 37 °C for 1 h. After washing, 100 µL of TMB substrate (KPL, USA) was added to each well, and the reaction was stopped with 2 N H_2_SO_4_. Absorbance was measured at 450 nm using a microplate reader. Antibody responses were expressed as sample-to-positive (S/P) ratios using the following formula: S/P = (OD sample − OD negative)/(OD positive − OD negative). Samples with an S/P ratio ≥ 0.4 were considered positive according to the previously validated cutoff value [32].

The positive control serum was obtained from PEDV-infected pigs experimentally confirmed by virus neutralization assay, whereas the negative control serum was collected from PEDV-naïve pigs with no history of infection or vaccination. Positive and negative control sera were included on each plate to ensure assay consistency. All samples were tested in duplicate, and assay performance was monitored using internal control sera as part of routine quality control procedures.

### 2.5. Serum Neutralization Test (SNT)

Serum samples were heat-inactivated at 56 °C for 30 min. Two-fold serial dilutions of each serum were prepared in duplicate and mixed with an equal volume of PEDV suspension containing 100 TCID_50_ per well. After incubation at 37 °C for 1 h, the mixtures were added to confluent Vero cell monolayers in 96-well plates and incubated for 1 h to allow virus adsorption. After removal of the inoculum, the cells were washed and overlaid with maintenance medium containing 10 µg/mL trypsin, and plates were incubated at 37 °C for 48 h. Cytopathic effect (CPE) was evaluated by microscopic examination. PEDV-positive and PEDV-negative control sera were included in each assay. Neutralizing antibody titers were expressed as the reciprocal of the highest serum dilution that completely prevented (100%) CPE in the infected cells.

### 2.6. Statistical Analysis

Data for ELISA S/P ratios (IgG and IgA) and serum neutralization titers (log_2_-transformed) were analyzed at each time point. Normality of the data was assessed using the Shapiro–Wilk test, and homogeneity of variances was evaluated using Levene’s test. The results indicated that the data were normally distributed and met the assumptions for parametric analysis. Therefore, group differences were analyzed using one-way analysis of variance (ANOVA) followed by Tukey’s honestly significant difference (HSD) post hoc test. Statistical analyses were performed using R software (version 4.1.2). The results are presented as mean ± SD, and differences were considered statistically significant at *p* ≤ 0.05.

## 3. Results

### 3.1. Clinical Observations and Safety Assessment

All vaccinated and control pigs remained clinically healthy throughout the study period. No abnormal clinical signs, including fever, diarrhea, reduced appetite, behavioral changes, or local injection site reactions, were observed following either primary or booster vaccination. No systemic or overt adverse reactions were detected in any group.

### 3.2. PEDV-Specific IgG Responses

**Figure 1:**
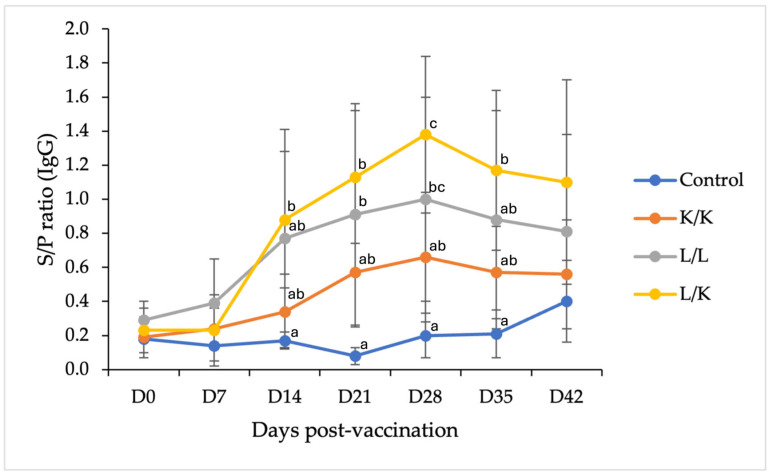
Serum PEDV-specific IgG responses in replacement gilts following different vaccination regimens. Serum IgG antibody levels were measured by ELISA and expressed as S/P ratios. Gilts were vaccinated with PBS (control), K/K, L/L, or L/K (*n* = 6 per group). Data are presented as mean ± SD at each sampling time point. Different superscript letters within the same time point indicate significant differences among groups (*p* < 0.05).

PEDV-specific IgG levels were measured by ELISA and expressed as S/P ratios (Figure 1). All vaccinated groups developed detectable IgG responses following primary immunization, whereas the PBS control group remained below the cut-off value throughout the study period. A pronounced increase in IgG S/P ratios was observed after the booster vaccination administered on day 14.

Among the vaccinated groups, the heterologous prime-boost regimen (L/K) exhibited the highest mean IgG responses, with elevated S/P ratios detected from day 21 onward and peak levels observed at day 28. Although IgG levels in the L/K group gradually declined after day 28, they remained numerically higher than those observed in the other vaccination groups through day 42. The homologous live vaccine group (L/L) also showed strong IgG induction, reaching peak levels at day 28, but its mean responses were numerically lower than those of the L/K group (Figure 1). In contrast, the inactivated vaccine group (K/K) induced only moderate IgG responses, which were significantly lower than those elicited by the live vaccine-containing regimens (L/L and L/K) at most post-booster time points.

Statistical analysis indicated that at days 21 and 28 post-vaccination, IgG S/P ratios in the L/K group were significantly higher than those in the K/K and PBS control groups (*p* ≤ 0.01). The L/L group also exhibited significantly higher IgG levels than the PBS group at day 28 (*p* < 0.05). However, no statistically significant differences were observed between the L/L and L/K groups at any time point. By day 42, IgG levels had declined in all vaccinated groups, and no significant differences were detected among the vaccination regimens. Detailed numerical data are provided in Supplementary Table S1.

### 3.3. PEDV-Specific IgA Responses

**Figure 2:**
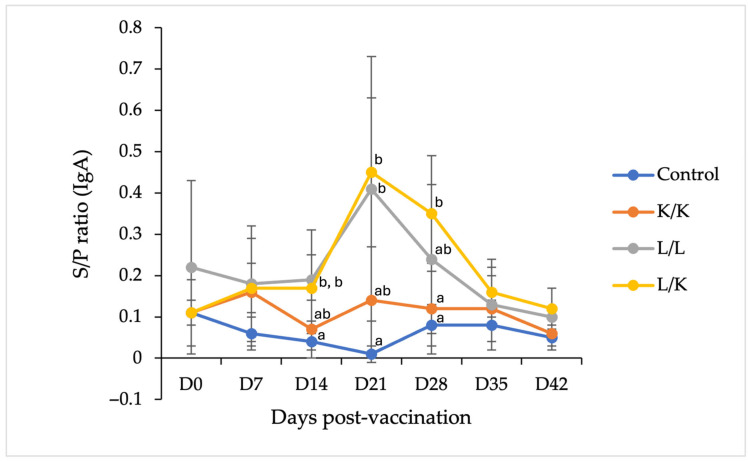
Serum PEDV-specific IgA responses in replacement gilts following different vaccination regimens. IgA levels were measured by indirect ELISA and expressed as S/P ratios. Pigs were vaccinated with PBS (control), K/K, L/L, or L/K (*n* = 6 per group). Data are presented as mean ± SD values at each time point.

Serum PEDV-specific IgA responses were generally lower in magnitude and more transient than IgG responses (Figure 2). Following the booster immunization, IgA S/P ratios in the L/K and L/L groups increased and transiently reached or exceeded the assay cutoff (S/P ≥ 0.4) at day 21, indicating a short-lived induction of serum IgA. The L/K regimen showed numerically higher peak IgA levels than the L/L group at day 21; however, this difference was not statistically significant.

After day 21, IgA responses declined rapidly, and S/P ratios in all vaccinated groups fell below the cutoff value by day 28 and remained low through day 42. Throughout the study period, pigs in the K/K and PBS control groups consistently maintained IgA S/P ratios below the cutoff, indicating minimal induction of serum IgA following the inactivated vaccine or in unvaccinated animals.

Statistical analysis indicated higher IgA S/P ratios in the L/L and L/K groups compared with the PBS control group at days 14 and 21. At day 28, IgA levels in the L/K group remained higher than those in the PBS and K/K groups; however, these values were below the assay cutoff. IgA S/P ratios were comparable among groups at later time points. Detailed numerical data are provided in Supplementary Table S2.

### 3.4. PEDV-Specific Neutralizing Antibody Responses

**Figure 3:**
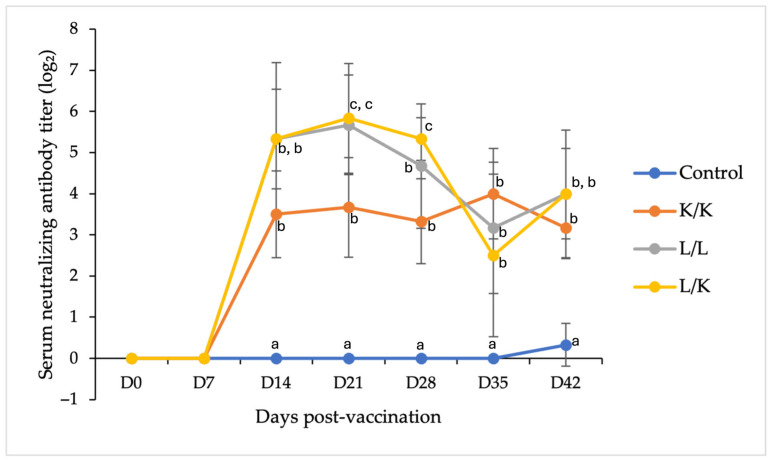
Serum-neutralizing antibody titers in replacement gilts following different vaccination regimens. Titers were measured by a serum neutralization test and expressed as log_2_ values. Pigs were vaccinated with PBS (control), K/K, L/L, or L/K (*n* = 6 per group). Statistical differences among groups at each time point were analyzed by one-way ANOVA with Tukey’s HSD test; different superscript letters indicate significant differences (*p* < 0.05).

Serum-neutralizing antibody titers were measured by a serum neutralization test and expressed as log_2_ values (Figure 3). No neutralizing activity was detected in any group at days 0 and 7, and pigs in the PBS control group remained seronegative throughout the entire experimental period. All vaccinated groups developed detectable neutralizing antibody titers following immunization.

The heterologous prime-boost (L/K) and homologous live vaccine (L/L) regimens induced the highest neutralizing antibody responses, with peak titers observed between days 21 and 28. At day 21 post-vaccination, neutralizing antibody titers in both the L/L and L/K groups were significantly higher than those in the K/K and PBS control groups (*p* < 0.001). At day 28, the L/K group exhibited significantly higher neutralizing antibody titers than both the K/K and L/L groups, indicating a stronger booster effect of the inactivated vaccine following live priming.

The K/K group developed moderate neutralizing activity, with titers increasing gradually and reaching peak levels at day 35; however, these titers were significantly lower than those in the L/K group at key post-booster time points. By days 35 and 42, neutralizing antibody titers had declined in all vaccinated groups but remained significantly higher than those in the PBS control group. The complete neutralization dataset is available in Supplementary Table S3.

## 4. Discussion

This study demonstrated that a heterologous prime-boost regimen consisting of a live-attenuated PEDV vaccine followed by an inactivated booster (L/K) induced strong systemic humoral immune responses in PEDV-naïve replacement gilts. This vaccination strategy generated the highest PEDV-specific serum IgG and neutralizing antibody titers and performed comparably to or better than homologous live (L/L) or inactivated (K/K) vaccination regimens. These findings are consistent with established principles of vaccine immunology indicating that heterologous prime-boost strategies can enhance immune priming, memory B-cell responses, and the durability of antibody responses compared with homologous vaccination [29,33,34].

The enhanced humoral immune responses observed in the L/K group likely reflect the complementary immunological properties of the two vaccine platforms rather than differences in adjuvantation, as the same adjuvant formulation was used across all vaccine groups. Live-attenuated PEDV vaccines are known to induce robust innate and adaptive immune activation during the priming phase, thereby establishing a strong immunological foundation for subsequent boosting [35]. In contrast, inactivated vaccines provide a stable and consistent antigenic stimulus that effectively amplifies pre-existing immune memory during booster immunization [25,26,27,33,36]. The heterologous combination employed in the L/K regimen may therefore synergize early immune activation with effective antibody boosting, resulting in enhanced magnitude and persistence of systemic humoral immunity. Similar advantages of heterologous prime-boost strategies have been demonstrated in influenza vaccination models, in which sequential immunization with antigenically distinct inactivated viruses elicited broader and more effective cross-protective antibody responses than homologous repeat vaccination [37]. Consistent with this interpretation, the antibody kinetics observed in the present study—characterized by an initial response following priming and a pronounced increase after booster vaccination—align with classical patterns of vaccine-induced humoral immune responses [25,26].

Although other heterologous combinations, such as an inactivated prime followed by a live-attenuated boost (K/L), could theoretically be evaluated, live priming is generally considered advantageous for effective immune activation because it can stimulate both innate and adaptive immune responses during the initial phase of vaccination. In contrast, priming with an inactivated vaccine may lead to comparatively lower initial immune stimulation, and subsequent boosting with a live-attenuated virus may introduce additional variables related to viral replication and safety. Therefore, the present study focused on vaccination regimens that are biologically rational and aligned with practical vaccination strategies.

Previous studies conducted in sows have reported that both live and inactivated PEDV vaccines can increase PEDV-specific IgG, IgA, and neutralizing antibody levels in serum, colostrum, and milk, which are associated with improved lactogenic protection of neonatal piglets [3,18,19,23,38]. However, the effectiveness of PEDV vaccination under field conditions remains variable, influenced by factors such as viral strain diversity, antigenic mismatch, vaccination timing, and parity [7,8,9,39,40]. A recent systematic review further emphasized the heterogeneity in immune responses and protective efficacy among different PEDV vaccine platforms [24].

Replacement gilts represent a practical and well-established pre-breeding model for comparing systemic immune responses between vaccination regimens when direct evaluation in pregnant sows is logistically challenging [30,31]. In the present study, serum IgA responses were generally low in magnitude and transient across all experimental groups, reflecting the limited capacity of parenteral vaccination to induce strong mucosal immune responses due to compartmentalization of the mucosal immune system [22]. Although transient increases in serum IgA were observed following booster vaccination, these responses rapidly declined and largely remained below the assay cutoff, underscoring their limited biological relevance in this experimental context. Accordingly, the immune responses measured in this model primarily reflect systemic immune priming rather than lactogenic or mucosal protection. While serum IgA may provide supportive information regarding systemic humoral activation, direct assessment of mucosal IgA in the intestine or mammary gland would be required to fully evaluate protective lactogenic immunity against PEDV [41].

Taken together, the findings of this study indicate that heterologous prime-boost vaccination with a live-attenuated PEDV vaccine followed by an inactivated booster elicits robust systemic humoral immune responses in replacement gilts. The enhanced serum IgG and neutralizing antibody responses observed with the L/K regimen highlight its potential advantages in immune priming compared with homologous vaccination strategies. Under commercial farm conditions, vaccinated gilts were routinely observed for general health status throughout the study period. No abnormal clinical signs or overt adverse reactions were detected following L/K vaccination, and no management concerns were noted, indicating acceptable field-level tolerability of this regimen in replacement gilts.

This study was conducted under commercial farm conditions using PEDV-naïve 16-week-old replacement gilts, enabling controlled comparison of homologous and heterologous vaccination regimens and clear evaluation of primary systemic humoral immune priming through PEDV-specific IgG and serum-neutralizing antibody responses. Establishing immunity at the pre-breeding stage represents a practical component of herd vaccination programs and provides a foundation for subsequent gestational boosting. However, because lactogenic immunity is the primary correlate of protection for neonatal piglets, this study did not assess mucosal antibodies in colostrum or milk, nor did it evaluate protective outcomes in suckling piglets. In addition, no virulent PEDV challenge was performed; therefore, the findings should be interpreted as evidence of comparative immunogenicity rather than direct protective efficacy. Further studies in pregnant gilts and sows, including assessment of colostrum and milk IgA and neutralizing antibodies as well as controlled challenge or field protection endpoints, are warranted to confirm translational relevance for piglet protection.

## 5. Conclusions

This study demonstrated that a heterologous prime-boost PEDV vaccination strategy using a live-attenuated vaccine followed by an inactivated booster induced strong systemic humoral immune responses in PEDV-naïve replacement gilts, characterized by high levels of PEDV-specific IgG and neutralizing antibodies. Although serum IgA responses were transient, the L/K regimen showed favorable immunogenicity compared with homologous vaccination regimens. Further studies in pregnant gilts and sows are required to determine whether this vaccination strategy can enhance lactogenic immunity and provide effective protection against PEDV in suckling piglets.

## Data Availability

The data presented in this study are available on request from the corresponding author.

## Supplementary Materials

The following supporting information can be downloaded at: https://www.mdpi.com/article/10.3390/vaccines14030231/s1, Table S1: Serum PEDV-specific IgG S/P ratios in pigs following different vaccination protocols at indicated time points post-vaccination; Table S2: Serum PEDV-specific IgA S/P ratios in pigs following different vaccination protocols at indicated time points post-vaccination; Table S3: Serum neutralizing (SN) antibody titers (log_2_) in pigs from different experimental groups at various time points post-vaccination.

## Abbreviations

The following abbreviations are used in this manuscript:

ASFV	African swine fever virus
CSFV	Classical swine fever virus
ELISA	Enzyme-linked immunosorbent assay
IACUC	Institutional Animal Care and Use Committee
K/K	Inactivated vaccine prime followed by inactivated vaccine boost
L/K	Live-attenuated vaccine prime followed by inactivated vaccine boost
L/L	Live-attenuated vaccine prime followed by live-attenuated vaccine boost
PBS	Phosphate-buffered saline
PCV2	Porcine circovirus type 2
PED	Porcine epidemic diarrhea
PEDV	Porcine epidemic diarrhea virus
PRRSV	Porcine reproductive and respiratory syndrome virus
RT-PCR	Real-time reverse transcription polymerase chain reaction
SN	Serum neutralization
S/P	Sample-to-positive
