# Supplementary material for: Comparative Humoral Immune Responses Induced by Live-Attenuated and Inactivated Porcine Epidemic Diarrhea Vaccines in Replacement Gilts
Source: Vaccines (Basel). 2026 Feb 28;14(3):231. doi: 10.3390/vaccines14030231 (PMC13030667; doi:10.3390/vaccines14030231)
Supplement: Supplementary file 1 [file vaccines-14-00231-s001.zip › vaccines-4144656-supplementary.pdf]

Supplementary Materials

Comparative Humoral Immune Responses Induced by Live-Attenuated and Inactivated Porcine Epidemic Diarrhea Vaccines in Replacement Gilts

Table S1. Serum PEDV-specific IgG S/P ratios in pigs following different vaccination protocols at indicated time points post-vaccination.

Day	Control (PBS)	K/K	L/L	L/K	*p*-value
D0	0.18 ± 0.11	0.19 ± 0.12	0.29 ± 0.11	0.23 ± 0.13	0.41
D7	0.14 ± 0.09	0.24 ± 0.12	0.39 ± 0.26	0.23 ± 0.21	0.16
D14	0.17 ± 0.05[a]	0.34 ± 0.22[ab]	0.77 ± 0.64[ab]	0.88 ± 0.40[b]	0.01
D21	0.08 ± 0.05[a]	0.57 ± 0.32[ab]	0.91 ± 0.65[b]	1.13 ± 0.39[b]	0.002
D28	0.20 ± 0.13[a]	0.66 ± 0.38[ab]	1.00 ± 0.60[bc]	1.38 ± 0.46[c]	< 0.001
D35	0.21 ± 0.14[a]	0.57 ± 0.27[ab]	0.88 ± 0.64[ab]	1.17 ± 0.47[b]	0.006
D42	0.40 ± 0.24	0.56 ± 0.32	0.81 ± 0.57	1.10 ± 0.60	0.08

Data are expressed as mean ± SD. Different superscript letters within the same row indicate significant differences among groups ($p < 0.05$). Statistical analysis was performed using one-way ANOVA at each time point, followed by Tukey's honestly significant difference (HSD) test.

Table S2. Serum PEDV-specific IgA S/P ratios in pigs following different vaccination protocols at indicated time points post-vaccination.

Day	Control (PBS)	K/K	L/L	L/K
D0	0.11 ± 0.03	0.11 ± 0.08	0.22 ± 0.21	0.11 ± 0.08
D7	0.06 ± 0.04	0.16 ± 0.13	0.18 ± 0.14	0.17 ± 0.06
D14	0.04 ± 0.02[a]	0.07 ± 0.07[ab]	0.19 ± 0.12[b]	0.17 ± 0.08[b]
D21	0.01 ± 0.02[a]	0.14 ± 0.13[ab]	0.41 ± 0.32[b]	0.45 ± 0.18[b]

Day	Control (PBS)	K/K	L/L	L/K
D28	0.08 ± 0.05[a]	0.12 ± 0.11[a]	0.24 ± 0.18[ab]	0.35 ± 0.14[b]
D35	0.08 ± 0.06	0.12 ± 0.08	0.13 ± 0.11	0.16 ± 0.06
D42	0.05 ± 0.03	0.06 ± 0.04	0.10 ± 0.07	0.12 ± 0.05

Data are presented as mean ± SD. Different superscript letters within the same row indicate significant differences among groups ($p < 0.05$) based on one-way ANOVA followed by Tukey's honestly significant difference (HSD) test. An S/P ratio ≥ 0.4 was considered positive. Although transient increases in S/P ratios were observed in some vaccinated groups, most values remained close to or below the positivity cutoff, indicating limited systemic IgA responses following vaccination.

Table S3. Serum neutralizing (SN) antibody titers (log2) in pigs from different experimental groups at various time points post-vaccination. Data are presented as mean ± SD.

Day	Control (PBS)	K/K	L/L	L/K	*p*-value
D0	0.00 ± 0.00	0.00 ± 0.00	0.00 ± 0.00	0.00 ± 0.00	–
D7	0.00 ± 0.00	0.00 ± 0.00	0.00 ± 0.00	0.00 ± 0.00	–
D14	0.00 ± 0.00[a]	3.50 ± 1.05[b]	5.33 ± 1.86[b]	5.33 ± 1.21[b]	< 0.001
D21	0.00 ± 0.00[a]	3.67 ± 1.21[b]	5.67 ± 1.21[c]	5.83 ± 1.33[c]	< 0.001
D28	0.00 ± 0.00[a]	3.33 ± 1.03[b]	4.67 ± 1.51[b]	5.33 ± 0.52[c]	< 0.001
D35	0.00 ± 0.00[a]	4.00 ± 1.10[b]	3.17 ± 1.60[b]	2.50 ± 1.97[b]	< 0.001
D42	0.33 ± 0.52[a]	3.17 ± 0.75[b]	4.00 ± 1.10[b]	4.00 ± 1.55[b]	< 0.001

Data are expressed as mean ± SD. Different superscript letters within the same row indicate significant differences among groups ($p < 0.05$). Statistical analysis was performed using one-way ANOVA at each time point, followed by Tukey's honestly significant difference (HSD) test.
